# A comparative study on the use of procalcitonin to distinguish between central fever and infectious causes of fever

**DOI:** 10.11604/pamj.2024.47.43.37617

**Published:** 2024-02-05

**Authors:** Iffat Khanum, Maheen Sattar Shoaib, Safia Awan

**Affiliations:** 1 Department of Medicine, Aga Khan University Hospital, Karachi, Pakistan; 2 Department Of Medicine, Jinnah Postgraduate Medical Centre, Karachi, Pakistan

**Keywords:** Procalcitonin, fever, infections, stroke, central nervous system

## Abstract

**Introduction:**

central fever is defined as elevated body temperature without any evidence of infection or drug reaction fever, and currently it has no definitive diagnostic criteria. The current study aims to assess the role of procalcitonin (PCT) in differentiating central fever from fever secondary to infections in patients with neurological insults.

**Methods:**

we conducted a retrospective study of patients admitted with a neurological insult (brain trauma, brain tumors and cerebrovascular accidents) in a tertiary care hospital. All patients who developed fever 48 hours after admission and had procalcitonin, C-reactive protein (CRP), and Erythrocyte sedimentation rate (ESR) done as part of fever evaluation were assessed to include in the study.

**Results:**

out of 70 patients who met inclusion criteria, 37 had infections identified and 33 had no source of infection. The mean age was 42.9 years (± 18) in the infectious group while 40.3 years (± 18.2) in the central fever group and there was male predominance in both groups. In the infectious group there were 25(67.6%) males vs. 12(32.4%) females while in non -infectious group, males vs. females were 18(54.5%) vs. 15(45.5%) and there was no difference in both group (p-value 0.26) Median procalcitonin (PCT) value was 0.09 ng/dl (IQR 0.05- 0.19) in patients with no identified cause of infection and 1.4 ng/dl (IQR 0.5-5.1) in patients with infections with a p-value of <0.001. Although CRP and ESR were low in patients with central fever as compared to those with infections, these differences did not reach statistical significance with p-value of CRP 0.18 and p-value of ESR 0.31 between two groups.

**Conclusion:**

PCT levels were low in patients with central fever and may be considered as a useful biomarker to differentiate between infectious fever from non-infectious fever in patients with brain injury. This can prevent unnecessary antibiotic use in patients without infection.

## Introduction

Fever is common in hospitalized patients with neurological insults, often attributed to underlying infections. However, research has shown that non-infectious causes account for approximately half of these cases [1,2]. Non-infectious causes include central fever, drug fever, and post-transfusion reactions [3,4].

Central Fever (CF) is characterized by elevated body temperature without evidence of infection or drug reaction [5]. It presents with rapid onset, fluctuating temperature, and poor response to antibiotics and antipyretics [6]. CF is believed to result from dysfunction in structures involved in temperature regulation, such as cutaneous thermal receptors, hypothalamus, midbrain, and spinal cord. Conditions like brain tumors, subarachnoid hemorrhages (SAH), intracerebral hemorrhages (ICH), and major neurosurgical procedures are commonly associated with CF [3,7]. While there is currently no definitive diagnostic criterion for CF, it should be considered in the differential diagnosis of febrile patients with brain insults. Many patients with fever in neurology and neurosurgical services are empirically treated with antibiotics and need comprehensive evaluation to reach the diagnosis [3,8]. Although microbiological cultures are considered as a diagnostic tool but they typically take 48 hours to produce initial results, costly and carry a risk of false negatives, particularly in patients who have received prior antimicrobial therapy [9,10]. The inappropriate use of antibiotics has led to the emergence of multi drug-resistant organisms (MDROs), increased healthcare costs, prolonged hospital stays, and higher mortality rate [11,12]. Early antibiotic use in non-infectious fever has also been associated with increased morbidity and mortality in this patient population [13,14].

Many inflammatory biomarkers including CRP, Cytokines i.e. Tumor necrosis factors (TNF), interleukin-6 (IL-6) and ESR aid in diagnosis of complex inflammatory and infectious disorders but lack specificity [15,16]. Procalcitonin (PCT), a precursor of calcitonin, is found to be high in bacterial infections and could be potential diagnostic and prognostic biomarkers to differentiate infectious from non-infectious etiologies of fever [17-19]. However, some studies have shown that PCT levels cannot reliably differentiate between infectious and central fever [20,21]. Fever in patients with brain injury is associated with prolonged ICU and hospital stay and worse overall outcomes [22]. Early Identification of cause of fever allows for tailored management and improved patient outcomes. The study aimed to evaluate the utility of PCT as a marker for central fever, which would enable the safe discontinuation of antibiotics despite the presence of persistent fever. This approach would help reduce the acquisition of MDROs, decrease healthcare costs, and shorten hospital stays.

## Methods

**Study design and setting:** we conducted a retrospective study of adult patients admitted with neurological insults from April 2019 to September 2019 at tertiary care hospital in Karachi, Pakistan. Established in 1985, this university hospital is JCI accredited, one of the largest tertiary care university hospitals (740 bedded), and caters to a variety of patients referred from all over country.

**Study population:** all adult patients with fever and central nervous system pathology like primary intracerebral hemorrhage, subdural hematoma (SDH), spontaneous and traumatic subarachnoid hemorrhage (SAH), ischemic stroke, traumatic brain injury (TBI), brain tumor and serum PCT levels available were evaluated for possible inclusion in the study. All the patients who were on antibiotics for prior infection within 15 days of the present episode of fever, inadequate data, viral infections like dengue fever, malaria, blood transfusion reaction and venous thrombo-embolisms were excluded.

**Sample size estimation:** all adult patients admitted with fever and neurological insult (CNS) admitted during study period at AKUH were evaluated for possible inclusion in study. The sample technique was convenient sampling. Previous study shows AUC 95% of PCT for predicting fever with 95% confidence level and 80% power, for detection of an effect of 10%, the required sample size is 114 participants.

**Data collection:** the data were collected from medical records using structured questionnaire and consisted of patient demographic details, primary neurological diagnosis, fever profile, laboratory and radiological investigations, length of hospital stay and outcome. Lab parameters including PCT, CRP and ESR along with total leukocyte count and neutrophil count on the day of onset of fever were recorded. Microbiological culture results i.e., cultures obtained from blood, urine, respiratory secretions, wound sites and cerebrospinal fluid were also recorded.

**Definitions:** fever was defined as a core body temperature of higher than 380C (F). Fever was considered infectious etiology on the basis of the physician´s clinical assessment as documented in medical records and/or on the basis of results from microbiological and radiological investigations. Central fever was defined as fever with no evidence of any source of infection in the presence of a neurological insult.

**Statistical analysis:** the analysis was performed using SPSS (Statistical Package of Social Sciences) version 19. Continuous variables with normal and non-normal distributions were reported as mean ± SD and median [inter-quartile range (IQR)], respectively. Prevalence (%) of demographic as well as clinical factors was assessed and divided into infectious group and non-infectious group. Continuous variables with normal distribution were analyzed using independent samples t-test, while those with skewed distribution were analyzed using Mann-Whitney U test. All p-values were based on two-sided tests and significance was set at a p-value less than 0.05.

**Ethical considerations:** the study received approval from the Ethical Review Committee (ERC) of the Aga Khan University Hospital (ERC no 2019-1145-3211). The requirement for informed consent was waived by hospital ERC as no personal identifiers were collected and data was anonymized. Confidentiality of data was strictly maintained as per hospital ERC policy.

## Results

**General characteristics of the study population:** out of 116 patients with CNS injury and fever, 70 patients met the inclusion criteria and were included in the study. Among these 70 patients, 37(52.8%) had fever secondary to an identifiable infectious etiology and 33(47.1%) were classified as central fever. The mean age was 42.9 years (±18) in the infectious group while 40.3 years (± 18.2) in the central fever group and there was male predominance in both groups. In the infectious group there were 25(67.6%) males versus 12(32.4%) females while in non-infectious group, males versus females were 18(54.5%) vs. 15(45.5%) and there was no difference in both group (p-value 0.26). The common causes of CNS injury in the study population were intra cerebral hemorrhage (30%), subdural hematoma (SDH, 16%), sub-arachnoid hemorrhage (SAH, 15%), traumatic brain injury (TBI, 14%), ischemic stroke (13%), and brain tumor (12%).

**Figure 1:**
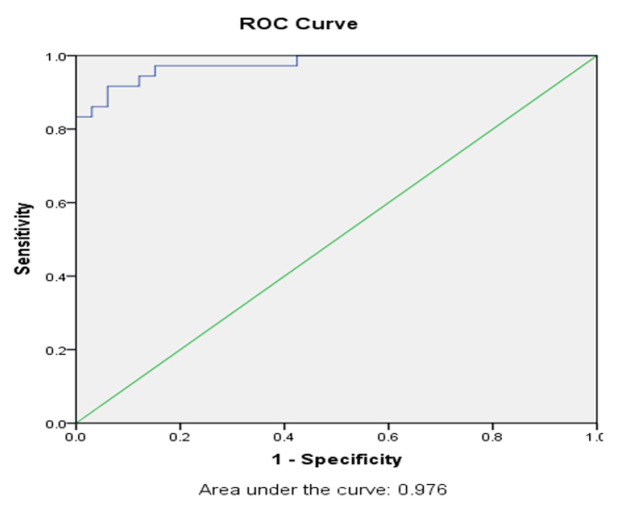
procalcitonin as a predictor for infection

**Relationship between biomarkers and fever origin:** no differences were observed in the fever pattern among the two groups, with the majority of patients (75.7%) having intermittent fever. Duration of fever was 6.5 (IQR 3-11.7) days and the maximum temperature recorded for both groups was 38.7°C (±0.5). The median procalcitonin (PCT) value among patients with central fever was 0.09 ng/ml (IQR 0.05-0.19) and patients with infections was 1.4 ng/ml (IQR 0.5-5.1) (p-value <0.001). Median C-reactive protein (CRP) levels in patients with fever secondary to infectious source was 29.5 mg/l (IQR 3.5-48.5), while median CRP levels among central fever patients were 5.7 mg/l (IQR 1.4-12). Similarly, the median erythrocyte sedimentation rate (ESR) was 70 mm/hour (IQR 19-111) in patients with infections and 49 mm/hour (IQR 15.5-71) in those with central fever. Although CRP and ESR were lower in patients with central fever compared to those with infections, these differences did not reach statistical significance with p-value of CRP 0.18 and p-value of ESR 0.31 between two groups. Neutrophil percentage was notably higher in the infectious fever group (83.5±5.4) vs central fever group (72.2±8.7) (p<0.001) (Table 1). ROC curve analysis showed that PCT was a good predictor for infection (AUC= 0.976, 95% confidence interval; 0.947-1.00) (Figure 1).

**Table 1:** laboratory results of patients with infectious versus non-infectious fever

parameters	Infection n=37	No-infection n=33	p-value
CRP	29.5 (3.5-48.5)	5.7 (1.4-12)	0.18*
ESR	70 (19-111)	49(15.5-71)	0.31*
PCT	1. 4 (0.5-5.1)	0.09 (0.05-0.19)	<0.001*
Leukocytes	13.7 (9.7-16.6)	11.6 (8.4-14.9)	0.06*
**Neutrophils**	**84.3(79.9-87.4)**	**72.3(66.5-77.6)**	<0.001*

CRP: C reactive protein; ESR: erythrocyte sedimentation rate; PCT: Procalcitonin; *Mann-Whitney U test

**Infectious etiologies and treatment outcomes:** out of the 37 patients with identified source of infection, pneumonia was the leading cause of single site infection (n=16), followed by bloodstream infections (n=5), urinary tract infections (UTI, n=2), CNS infections (n=2), and wound site infections (n=2), while 10 patients had multiple site infections (Figure 1). A total of 65 out of 70 patients received antibiotics, including 36 (97.3%) patients from the infectious fever group and 29 (88%) patients from the central fever group. Antibiotics were discontinued early in only 4 cases of central fever (Table 2). No significant difference was found in the length of hospital stay among the two groups. The overall mortality rate in this study group was 11.4%. The median PCT among discharged patients was 0.30 (0.08-1.5), while patients who died had a slightly higher median PCT of 0.36 (0.17-15.3), but this difference was not statistically significant (p=0.28) (Table 3).

**Table 2:** antibiotics used in febrile patients with neurological insult

Antibiotics	Infectious n=37	non-infectious n=33
Ceftriaxone	4 (10.8%)	8 (24.2%)
Vancomycin	23 (62.2%)	18 (54.5%)
Meropenem	25 (67.6%)	18 (54.5%)
Piperacillin/Tazobactam	15 (40.5%)	13 (39.4%)
Ampicillin	2 (5.4%)	1 (3.0%)
Gentamicin	-	2 (6.1%)
Linezolid	2 (5.4%)	-
Colistin	13 (35.1%)	8 (24.2%)
Amikacin	1 (2.7%)	

**Table 3:** comparison between survivor and non-survivor

parameters	Death n=8	Discharge n=62	p-value*
ESR	112	51 (18.2-73)	0.15
PCT	0.36 (0.17-15.3)	0.30 (0.08-1.5)	0.28
Leukocytes	13.89 (9.4-14.8)	13.1 (8.9-15.7)	0.99

* Mann-Whitney U test; CRP: C reactive protein; ESR: erythrocyte sedimentation rate; PCT: Procalcitonin

## Discussion

In this retrospective study, we assessed the role of procalcitonin (PCT) compared to CRP and ESR in differentiating central fever from infectious fever in patients with neurological insults. We found that PCT, CRP and ESR levels were low in patients with central fever and high in patients with infectious fever. Importantly, PCT demonstrated the highest accuracy in distinguishing central fever from infection-related fever. Our key finding is that low levels of PCT may aid in ruling out infection in neurocritical care patients, thus allowing for early diagnosis of central fever and reducing unnecessary antibiotic use. This is a relevant clinical implication as the misuse of antibiotics, particularly in hospital settings, has led to an alarming rise in multidrug-resistant bacteria. PCT is present in undetectable concentrations in normal healthy individuals [17,18]. It is considered as a specific biomarker for bacterial infections and can be utilized safely for the escalation of antibiotics in patients with sepsis [23]. However, inadequate prior data exists for the utility of PCT in differentiating central fever from fever from infectious sources in patients with neurological injury [3].

Our results align with previous studies such as Kara *et al*., who found that median PCT levels of patients with intra cerebral hemorrhage (ICH) with infectious and central fever were 4 (IQR 0.9-11) ng/ml and 0.1 (IQR 0.1-0.4) ng/ml respectively [24]. Similarly, another study by Gautam-Goyal *et al*. revealed that PCT levels were high in patients with ICH and infections [25]. However, conversely, Halvorson *et al*. reported that PCT was a poor indicator of infectious fever in patients with neurological injury, highlighting the ongoing debate in the current literature and the need for further investigations [20].

The identification of central fever in patients admitted to a neurological intensive care unit (ICU) poses a significant challenge for physicians, primarily due to the absence of clear diagnostic criteria or a definitive definition and lack of reliable treatment modalities [3]. After an acute brain injury, CF often develops within 72 hours of admission. CF is considered as diagnosis of exclusion. Literature suggests that in critically ill patients with neurological insults, fever within 72 hours of hospitalization, normal chest radiographs, and negative microbiological investigations can serve as predictors of central fever [4]. Although these predictors contribute to the diagnosis of central fever, the need for a standardized diagnostics criteria remains. Our study presents promising findings that contribute towards the development of such criteria, bridging the gap in the identification and classification of central fever in this patient population.

Numerous biomarkers, such as interleukin 6 (IL 6), interleukin 2 (IL 2), CRP, and PCT, have been extensively studied in an effort to distinguish infections from non-infectious causes of fever in sepsis patients, which could potentially aid in optimizing the use of antibiotics [26]. However, the efficacy of these biomarkers for the diagnosis of central fever has not been firmly established yet, as robust scientific evidence is still needed to support their utility.

In our study, while elevated levels of erythrocyte sedimentation rate (ESR) and CRP were observed in patients with infections, the results did not demonstrate the effectiveness of these inflammatory markers in differentiating central fever from fever caused by infections. This is an important distinction to make, as CRP, a major acute phase reactant protein, remains elevated in various types of inflammatory processes. Consequently, it cannot be solely relied upon to differentiate infectious and noninfectious fever in neurocritical patients.

In contrast, a study conducted by Fluri *et al*. suggested that the combination of well-established inflammatory markers, such as white blood cell count (WBC) and CRP, along with biomarkers of bacterial infection like PCT, could prove useful in estimating the presence of infection following a stroke [27]. This finding highlights the potential benefits of employing a combination of biomarkers to improve diagnostic accuracy in differentiating the various causes of fever in neurocritical patients.

Furthermore, our study demonstrated that there was no difference in intensity, pattern, and duration of fever between the infectious and central fever groups, which contrasts with previous study, suggesting central fever is constant in nature with no diurnal deviations, plateau-like i.e. no fever spikes and resistant to antipyretic medications [3]. High PCT value can predict mortality in neuro critical patients. The overall mortality was 11% in our study group and the median PCT value was similar between survivors and non-survivors in our study. Studies had found that PCT levels did not correlate with mortality and other neurological outcomes. High PCT and CRP values at admission were an independent predictor of mortality in patients with acute ischemic stroke [28].

Regarding the limitations of our study, it should be noted that this was a single-center, retrospective study with a relatively small sample size. Additionally, we only considered the first episode of fever after hospital admission, while some patients with prolonged stay might have experienced multiple episodes of fever due to various causes. Finally, we did not have serial monitoring of biomarkers, including PCT levels, ESR, and CRP, which could have provided additional information.

Procalcitonin was identified as a reliable biomarker for the diagnosis of central fever in this retrospective single-center analysis, although the results might not accurately reflect the diversity and characteristics of the wider population. Healthcare environments or patient demographics may have a different impact on procalcitonin accuracy as a diagnostic of central fever in different group of patients. In comparison to retrospective or single-center research, well-designed prospective, randomized, multi-center studies typically offer higher evidence for external validity and generalizability.

## Conclusion

The misuse of antibiotics especially in hospital settings has led to an alarming rise in multidrug resistant bacteria. Despite extensive research, there is limited advancement in earlier diagnosis of infection. The findings in our study suggest that low levels of PCT can successfully rule out infection in neurocritical patients, leading to an early diagnosis of central fever and thus minimizing antibiotic use in such patients. Further studies including clinical trials are needed to validate the cut-off values of procalcitonin levels in central fever and the use of procalcitonin-guided antibiotic therapy.

### What is known about this topic

- *Fever is a prevalent symptom observed in patients experiencing neurological insults; although it can be attributed to infections, it is crucial not to overlook central fever as a significant contributing factor;*
- *Antibiotic treatment is usually started in the presence of fever before the confirmation of the presence of infection;*
- *CRP, ESR and procalcitonin are inflammatory biomarkers commonly raised in infections*.

### What this study adds

- *Low levels of serum procalcitonin levels can be used in early phase of illness to rule out bacterial infections in patients with acute neurological insult;*
- *Serum procalcitonin is more accurate in ruling out infection as compared to CRP and ESR;*
- *Early identification of central fever will enable timely discontinuation of antibiotics, thereby reducing the risk of antibiotic overuse*.
